# Comparing six mathematical link function models of the antifeedant activity of lesser grain borer exposed to sub-lethal concentrations of some extracts from *calotropis procera*

**DOI:** 10.1080/21655979.2019.1641399

**Published:** 2019-07-19

**Authors:** Elhadi E. Elamir, Abdulrhman A. Almadiy, Gomah E. Nenaah, Abdullah A. Alabas, Hajer S. Alsaqri

**Affiliations:** a Department of Mathematics, Najran University, Najran, Kingdom of Saudi Arabia; b Department of Biology, Najran University, Najran, Kingdom of Saudi Arabia

**Keywords:** Cauchy model, Complementary log-log model, feeding deterrence, logistic model, log-log model, Probit model

## Abstract

In the present study, Probit, Cauchy Fractional and three types of Log methods, i.e., Logit, Log-log, and Complementary log-log were employed to model the feeding deterrence of the lesser grain borer, *Rhyzopertha dominica* (F) (Coleoptera: Bostrichidae), when fed latex protein, crude flavonoid fraction, 3-O-rutinosides of quercetin, kaempferol and isorhamnetin, isolated from *Calotropis procera* (Ait.) (Gentianales: Asclepiadaceae). A nutritional study with treated flour discs at sub-lethal concentrations indicated that the tested natural products negatively affected the feeding behavior of the lesser grain borer, causing high feeding deterrent indices. Our results assure that Probit, Logit and Clog-log model the feeding deterrent indices with high goodness of fit. The models aim to support the management of the test insect when fed grains treated with sub-lethal doses of the tested phytochemicals in order to develop a viable, precise and long-term strategy to minimize the excessive reliance on the chemical pesticides currently in use.

## 1 Introduction

Insects of stored-products are serious pests of dried, stored and durable agricultural commodities. These pests are, in most cases, small-bodied and have a high reproductive potential. Therefore, they are easily concealed within grains and have been easily spread across the world. Once established in a commodity, they are usually difficult to control. Among all the biotic factors, insect pests cause (30%–40%) losses in stored grains [1].

The lesser grain borer, *Rhyzopertha dominica* is one of the most destructive primary pests of stored grains. This pest attacks the intact grain, where its larvae feed and develop inside the kernel. Synthetic pesticides such as organophosphates, carbamates, pyrethroids, and neonicotinoids and also, fumigants such as phosphine and methyl bromide are the major methods available for the protection of stored products against insect infestations [2,3]. Although highly effective, chemical insecticides are facing threats due to the high cost of application, the development of insect resistance, the toxic effects against non-target organisms and the negative environmental impacts [4–7]. As a result, researchers are seeking less hazardous strategies to combat stored grain insects. One of these approaches is the use of secondary metabolites from plants as pest control agents which have the advantage of providing novel modes of action that can reduce the risk of cross-resistance as well as offering new leads for the design of eco-friendly, less hazardous target-specific molecules [8–10].

In this context, control of insect pests using natural antifeedants is of crucial importance in the search for new and safe methods for pest control. In these control options, insects are usually subjected to sub-lethal doses of the test compounds leading to fewer hazards to non-target organisms, users and the ecosystem, to meet all standards required by the Environmental Protection Agency (EPA) and related organizations. Mathematical models provide a convenient medium for scientists to experiment with different empirical problems and obtain potentially important insights into the problems being studied. Mathematical biology is among the most exciting modern applications of mathematics. The increasing use of mathematics in biology is inevitable as biology becomes more quantitative. For the mathematician, biology opens up new and exciting branches, while for the biologist, mathematical modeling offers another research tool commensurate with a new powerful laboratory technique but only if used appropriately and its limitations are recognized. It is well known that estimating parameters based on measured empirical data is a critical issue in biosecurity models. However, there are many problems of quantitative inference in biological research concerning the relationship between a stimulus (e.g. treatment with phytochemicals) and a binomial response (e.g. anti-feeding effect). In this regard, mathematical models can provide a relatively fast, accurate and inexpensive way to project the consequences of different assumptions about the merits of various pest management options [11]. Good-fitting models have several benefits; it means good assessment of values where no observations are available and more precise summarization of relationships among two or more variables. In general, it means good data visualization. Therefore, improvements in such simulation models are urgently required. We will use the relative square errors ($L_{2}$) as a measure of goodness of fit of the proposed models;

$$L_{2}=\frac{\sum_{i=1}^{n}{\left(O_{i}-P_{i}\right)}^{2}}{\sum_{i=1}^{n}O_{i}^{2}},$$

where $O_{i}$ are the observed points and $P_{i}$ are the predicted points. Our main data are obtained as in [16], from the author.

A binomial generalized linear model, with a link function [12] such as the Probit, Logit or Cauchy functions is usually used to analyze the empirical biological data [13–15]. In the present study, we employ these models. Moreover, we suggest using Log-log, Clog-log and Fractional models with them. To the knowledge of the authors, no study has been published on this topic. These models help to develop a viable, precise and long-term strategy to support the management of the lesser grain borer, when fed grains treated with sub-lethal doses of latex proteins and bioflavonoids isolated from *C. procera* (Ait.) growing in southern Saudi Arabia in order to minimize the excessive reliance on the chemical pesticides currently in use.

## 2 Materials and methods

### 2.1 Test insect

A culture of *R. dominica* reared in the Department of Biology, College of Science and Arts, Najran University, Saudi Arabia was used in the current study. Insects were maintained in the dark in a growth cabinet under a constant temperature 30 ± 2°C and 68 ± 5% relative humidity.

### 2.2 Preparation of the test plant and extraction of test compounds

*C. procera* was collected from the pre-desertic region around Najran Province, Saudi Arabia. The Botanists of Biology Department, College Arts and Sciences, Najran University, Saudi Arabia authenticated a fresh sample of the plant. Following the same procedure as in [16]. The leaves were air-dried for 8 days in the shade at (28–32 °C daytime temperatures). The dried leaves were powdered mechanically using an electric blender (Braun Multiquick Immersion Hand Blender, B White Mixer MR 5550 CA, Germany). Powdered samples were maintained in tightly closed dry bags for extraction of the phytochemicals. The crude latex was collected from the healthy aerial parts of *C. procera* [17]. After being centrifuged in a bench centrifuge (5000 g at 4°C for 10 min), the soluble phase of the latex was filtered and dialyzed for 60 h (8°C) using membranes of 8000 molecular weight cut-off. Dialysis water was renewed three times daily and precipitate, comprising rubber was discarded. After dialysis, the samples were centrifuged again and the soluble phase, comprised almost entirely of soluble latex proteins was freeze-dried. The clean supernatants, laticifer proteins were lyophilized and dissolved in an appropriate solvent for subsequent bioassays. Flavonoids of *C. procera* were extracted from the leaf methanolic extract according to Sharififar et al. [18]. Purification of isolated compounds was made on Sephadex LH-20 column using 70% methanol to afford 3-O-rutinosides of quercetin, kaempferol, and isorhamnetin. Structure of compounds was confirmed by correlating with melting points, acid hydrolysis, and their spectral data.

### 2.3 Anti-feeding activity

The flour disc bioassay [19,20] using test concentrations ranging between 0.313 and 5.0 mg/mL of each test product or acetone only (control) was used to determine the effect of *C. procera* phytochemicals on the nutritional parameters of *R. dominica*. Feeding deterrent percentage (FDI) was calculated as described by Farrar et al. [21], and modified by Huang and Ho [19], as follows: FDI = [(C_c_ – C_t_)/C_c_] × 100, where C_c_ is the consumption of control discs and C_t_ is the consumption of treated discs, as the control and treated discs were placed in separate vials in no-choice tests.

## 3. Mathematical models

Mathematical modeling is a process by which a real-world problem is described by a mathematical formulation. The structural form of a model describes the patterns of association and interaction. The sizes of the model parameters determine the strength and importance of its effects. Accordingly, the model’s predicted values smooth the data and provide improved estimates of the expected values at possible explanatory variable values. In the present study, we employ Probit, Logit or Cauchy and moreover, Log-log, Clog-log and Fractional models to model the Feeding deterrence index of *R. dominica* exposed to some sub-lethal amounts of *C. procera* extracts.

### 3.1 Probit model

The Probit model is used to model dichotomous or binary outcome variables. In the Probit model, the inverse standard normal distribution of the probability is modeled as a linear combination of the predictors. Probit analysis is used to analyze many kinds of binomial response experiments especially, analysis of dose-response and toxicology. The Probit link function $\phi \left(P\right)=Y-5$ is the inverse cumulative distribution function associated with the standard normal distribution [22,23];

(1) $$P=\varphi^{-1}\left(Y-5\right)=\int_{-\infty }^{Y-5}\left(\frac{e^{-\frac{1}{2}u^{2}}}{\sqrt{2\pi }}\right)du,$$

where *P* is the actual FDI. Note that, adding five to $\varphi \left(P\right)$ just ensures all *Y* values are positive in practice [13].

### 3.2 Logit model

The Logit regression model is used to model dichotomous or binary outcome variables. Its shape is remarkably similar to the normal distribution but has the advantage of a closed form expression. The shape of this model is closed to the Probit model, especially from p=0.1 to p=0.9 but it has a heavier tail. For this model, the canonical link function [12] is given in the following form;

$Y=Logit\left(P\right)=\ln\frac{P}{1-P}$, hence $P=\frac{e^{Y}}{1+e^{Y}}.$ (2)

### 3.3 Log-log model

Another choice of the link function is called the Log-log model, with a simple link function [14];

(3) $$Y=-ln\left(-\ln P\right),$$

and thus;

(4) $$P=e^{-e^{-Y}},$$

for Log-log model P approaches 0 sharply but approaches 1 slowly.

### 3.4 Complementary log log (clog-log)

An alternative model to the Log-log model is called the Complementary log-log model, with simple link function [15];

(5) $$Y=ln\left(-ln\left(1-P\right)\right),$$

which is the inverse of the CDF of the extreme value (or log-Weibull) distribution, with CDF

(6) $$F\left(Y\right)=1-e^{-e^{Y}}.$$

### 3.5 Cauchy model

The link function of the Cauchy model takes the form [11];

(7) $$Y=\tan\left[\pi \left(P-\frac{1}{2}\right)\right].$$

The cumulative distribution function (CDF) of the Cauchy distribution, whose curve is sigmoid likes the probit and logistic curves, is as follows;

(8) $$P=\frac{1}{\pi }\arctan\left(Y\right)+\frac{1}{2}.$$

### 3.6 Fractional model

In this model, the link function takes the form;

(9) $$Y=\frac{P}{1+a_{f}P}$$

and, hence;

(10) $$P=\frac{Y}{1-a_{f}Y},$$

with parameter $a_{f}$ which minimizes the relative square errors ($L_{2}$) by solving the following equation;

(11) $$\frac{\partial L_{2}}{\partial a_{f}}=0.$$

The values of $a_{f}$ are as follows:

For Latex protein, $a_{f}=0.8180$, for Crude flavonoids, $a_{f}=1.1586$, for Quercetin, $a_{f}=2.4415$, for Kaempferol, $a_{f}=2.6963$, for Isorhamnetin, $a_{f}=3.7045$

### 3.7 Optimal procedure

This procedure aims to select the nearest predicted points to the observed ones. Suppose we have *N* predicted points and *M* models. Let $P_{i}^{j}$ be the predicted points, i=1,…,N,denotes the point number, j=1,…,M, denotes the model number, let $O_{i}$ be the observed points, and $P_{i}^{*}$ be the optimal points which are the nearest to $O_{i}$.

- If $P_{i}^{j}-O_{i}=0$ then $P_{i}^{*}=P_{i}^{j},i=1,\ldots ,N.$
- If $P_{i}^{j}-O_{i}>0$ for all j=1,…,M. Then $P_{i}^{*}=\min P_{i}^{j}$.
- If $P_{i}^{j}-O_{i}<0$ for all j=1,…,M. Then $P_{i}^{*}=\max P_{i}^{j}$.
- If $P_{i}^{j}-O_{i}>0$ for some points j=1,…,S;S=1,…,M. Then $P_{i}^{\left(1\right)}=\min P_{i}^{j}$,

and if $P_{i}^{j}-O_{i}<0$ for the other points j=S+1,…,M. Then $P_{i}^{\left(2\right)}=\max P_{i}^{j}$,

and $P_{i}^{\left(3\right)}=\frac{P_{i}^{\left(1\right)}+P_{i}^{\left(2\right)}}{2}$, then $P_{i}^{*}=P_{i}^{\left(k\right)}$ which minimizes $\left|P_{i}^{\left(k\right)}-O_{i}\right|,k=1,\ldots ,3.$

According to this procedure we computed the optimal predicted points $P_{i}^{*}$ for all models in Table 2

**Table 1.** Feeding deterrence index (FDI) of *R. dominica* exposed to *C. procera* extracts-treated food at sub-lethal concentrations, *t* = 3 days.

Concentrations (mg/mL)	0.313	0.625	1.250	2.500	3.750	5.000
Latex protein	0.102	0.305	0.561	0.859	0.930	**1.000**
Crude flavonoids	0.086	0.204	0.390	0.592	0.726	0.862
Quercetin-3-O-rutinoside	0.050	0.091	0.185	0.322	0.420	0.533
Kaempferol-3-O-rutinoside	**0.000**	0.088	0.185	0.284	0.405	0.491
Isorhamnetin-3-O-rutinoside	**0.000**	0.035	0.097	0.215	0.331	0.429

**Table 2.** **(a)**: Observed and predicted FDI against extracts concentrations using Probit, Logit, Log-log, Clog-log, Cauchy, Fractional, and Optimal models, *t* = 3 days. **(b)**: Observed and predicted FDI against extracts concentrations using Probit, Logit, Log-log, Clog-log, Fractional, Cauchy and Optimal models, *t* = 3 days.

Extractions	Concentrations	0.313	0.625	1.250	2.500	3.750	5.000
(a)							
Latex protein	Observed	0.102	0.305	0.561	0.859	0.930	**1.000**
	Probit	0.100	0.301	0.594	0.841	0.925	0.961
	Logit	0.103	0.293	0.598	0.843	0.919	0.951
	Log-log	0.074	0.343	0.644	0.835	0.898	0.929
	Clog-log	0.116	0.265	0.539	0.855	0.963	0.992
	Cauchy	0.114	0.234	0.638	0.854	0.896	0.914
	Fractional	0.136	0.295	0.498	0.762	0.958	1.000
	Optimal	0.102	0.301	0.567	0.855	0.925	1.000
Crude flavonoids	Observed	0.086	0.204	0.390	0.592	0.726	0.862
	Probit	0.079	0.203	0.403	0.632	0.752	0.822
	Logit	0.083	0.196	0.395	0.637	0.757	0.825
	Log-log	0.053	0.216	0.450	0.660	0.753	0.806
	Clog-log	0.095	0.189	0.356	0.602	0.759	0.855
	Cauchy	0.098	0.154	0.314	0.668	0.793	0.841
	Fractional	0.089	0.211	0.368	0.579	0.740	0.878
	Optimal	0.086	0.203	0.395	0.591	0.740	0.867
Quercetin-3-O-rutinoside with Log transformation	Observed	0.050	0.091	0.185	0.322	0.420	0.533
	Probit	0.043	0.099	0.196	0.335	0.430	0.501
	Logit	0.047	0.096	0.186	0.328	0.433	0.512
	Log-log	0.036	0.103	0.213	0.348	0.430	0.487
	Clog-log	0.050	0.095	0.177	0.318	0.433	0.528
	Cauchy	0.058	0.079	0.125	0.269	0.501	0.680
	Fractional	0.039	0.105	0.194	0.320	0.421	0.512
	Optimal	0.050	0.095	0.186	0.320	0.421	0.528
Quercetin-3-O-rutinoside without Log transformation	Observed	0.050	0.091	0.185	0.322	0.420	0.533
	Probit	0.079	0.096	0.138	0.257	0.414	0.587
	Logit	0.079	0.094	0.132	0.246	0.412	0.601
	Log-log	0.075	0.096	0.147	0.276	0.421	0.560
	Clog-log	0.080	0.094	0.129	0.235	0.406	0.636
	Cauchy	0.076	0.083	0.100	0.168	0.393	0.753
	Fractional	0.084	0.102	0.143	0.248	0.401	0.648
	Optimal	0.075	0.089	0.147	0.276	0.421	0.560
(b)							
Kaempferol-3-O-rutinoside	Observed	**0.000**	0.088	0.185	0.284	0.405	0.491
	Probit	0.034	0.083	0.174	0.310	0.406	0.479
	Logit	0.044	0.088	0.171	0.305	0.406	0.483
	Log-log	0.020	0.075	0.180	0.320	0.409	0.470
	Clog -log	0.051	0.094	0.170	0.298	0.402	0.489
	Cauchy	0.087	0.114	0.163	0.269	0.388	0.502
	Fractional	0.011	0.075	0.163	0.295	0.408	0.514
	Optimal	0.011	0.088	0.180	0.282	0.406	0.489
Isorhamnetin-3-O-rutinoside with Log transformation	Observed	**0.000**	0.035	0.097	0.215	0.331	0.429
	Probit	0.007	0.030	0.094	0.225	0.336	0.424
	Logit	0.014	0.037	0.093	0.217	0.331	0.428
	Log-log	0.002	0.021	0.094	0.238	0.341	0.417
	Clog -log	0.017	0.041	0.094	0.211	0.326	0.432
	Cauchy	0.044	0.058	0.085	0.154	0.270	0.450
	Fractional	0.000	0.041	0.110	0.218	0.316	0.413
	Optimal	0.000	0.034	0.094	0.214	0.331	0.428
Isorhamnetin-3-O-rutinoside without Log transformation	Observed	**0.000**	0.035	0.097	0.215	0.331	0.429
	Probit	0.052	0.064	0.094	0.181	0.308	0.464
	Logit	0.057	0.067	0.094	0.175	0.303	0.472
	Log-log	0.043	0.057	0.092	0.191	0.317	0.450
	Clog -log	0.061	0.071	0.096	0.172	0.298	0.484
	Cauchy	0.065	0.070	0.083	0.127	0.252	0.622
	Fractional	0.063	0.076	0.105	0.181	0.296	0.487
	Optimal	0.043	0.057	0.096	0.191	0.317	0.450

In all previous link-functions, *P* is the actual FDI (0≤P≤1) and Y is the transformed FDI, which may depend on time *t*, concentration *C* and their interaction (i.e., *C.t*) to get a 4- parameter model;

(12) $$Y=a+b_{1}\log\left(t\right)+b_{2}\log\left(C\right)+b_{3}\log\left(t\right)\log\left(C\right).$$

If we omit the influence of the interaction between *C* and *t*, we get a 3-parameter model in the form;

(13) $$Y=a+b_{1}\log\left(t\right)+b_{2}\log\left(C\right).$$

If the independent data do not depend on *C* or *t* separately, but on the product *C.t*, the parameters *b*_1_ and *b*_2_ can be merged into a single parameter *b*, and we get the 2-parameter model, which we will use here, in the form;

(14) $$Y=a+b\log\left(Ct\right)$$

For the un-transformed logarithmic model;

(15) Y=a+b.C.t.

### 3.8 Generalized inverse matrix approach

It is known that any matrix has an inverse only if it is square, and even then only if it is nonsingular. However, when we use the previous models, we usually get an over-determined system of linear equations in *a, b*_1_, *b*_2_, *b*_3_ in the form;

(16) $$Y_{i}=1.a+\left(\log t_{i}\right).b_{1}+(\log C_{i}).b_{2}+\left(\log t_{i}\right)\left(\log C_{i}\right).b_{3},\;i=1..N$$

Or, in the matrix form;

(17) Y1 Y2...YN=1 1...1logt1 logt2...logtNlogC1 logC2...logCNlogt1 logt2   logtNlogC1 logC2   logCNa b1 b2 b3

Now, for *N* observations, let the rectangular system (17) be in the form AX=Y where *A* is N×4 matrix. Then, $A^{T}AX=A^{T}Y$ hence $X=(A^{T}A{)}^{-1}A^{T}Y$ or $X=A^{+}Y$ where $A^{+}=(A^{T}A{)}^{-1}A^{T}$ is the generalized inverse matrix of *A* (or Moore–Penrose pseudo-inverse) [24], provided that $A^{T}A$ is non-singular. Note that, if *A* is non-singular then $A^{+}=A^{-1}$. Even if *A* is not full ranked [15], there still exists $A^{+}$ where the linear system has a solution $A^{+}y$ with minimum $L_{2}$.

### 3.9 Perturbation technique

Perturbation analysis examines the response of a model to changes in its parameters. We notice some zero observations in Table 1 and that some observations take their almost value (i.e., 1). At these values the link-functions of some models become undefined. The authors usually change the value 0 by 0.0001 and the value 1 by 0.9999 but it is not the case that the smallest changes of these values the smallest $L_{2}$ error. Therefore, we change these values by a small perturbed parameter ϵ. So, the value 0 becomes ϵ and the value 1 becomes (1- ϵ), and then, the relative square error $L_{2}$ becomes a function of the variable ϵ. Hence, we use the well-known Newton-Raphson technique to get ϵ which minimizes $L_{2}$, by solving the equation $\frac{dL_{2}\left(\epsilon \right)}{d\epsilon }=0,$ using the iterative formula [25], we get the following;

(18) $$\epsilon_{i+1}=\epsilon_{i}-\frac{L_{2}^{\prime }\left(\epsilon_{i}\right)}{L_{2}^{{\;}^{\prime \prime }}\left(\epsilon_{i}\right)}$$

where $L_{2}^{\prime }\left(\epsilon \right)$ and $L_{2}^{{\;}^{\prime \prime }}\left(\epsilon \right)$ are the first and the second derivative of $L_{2}\left(\epsilon \right)$ with respect to ϵ. It will give the best choice of ϵ.

## 4 Results

In this study, we used Probit, Logit, Log-log, Clog-log, Cauchy and Fractional methods to model the FDI of *R. dominica* using sub-lethal concentrations of latex proteins and flavonoids of *C. procera*. Evaporation of the extraction solvents gave petroleum ether (12.2 g), chloroform (6.7 g) and methanol extract (33.1 g). By correlating with melting points and spectral data of literature values, kaempferol-3-O-rutinoside (40.6 mg), isorhamnetin-3-O-rutinoside (27.8 mg) and quercetin-3-O-rutinoside (21.7 mg), were identified and isolated from the crude flavonoid fraction (214.2 mg) of MeOH extract.

Table 1 gives the percentage of feeding deterrence index (FDI) of *R. dominica* exposed to sub-lethal concentrations of C. *Procera* extracts. In an overview of this table, one can recognize that the FDI influence of the extracts on *R. dominica* gradually decreases from Latex Protein to Isorhamnetin-3-O-rutinoside respectively, and normally, increases with increasing in concentration ratios.

Table 2 lists the observed and predicted FDI against concentrations using Probit, Logit, Log-log, Clog-log, Cauchy and Fractional models for log transformed concentrations and time as explanatory variables, also, for untransformed variables for Quercetin-3-O-rutinoside and Isorhamnetin-3-O-rutinoside.

Table 3 gives the values of ϵ and $L_{2}$ for all extracts and all used models with log transformed variables, and also, for Quercetin-3-O-rutinoside and Isorhamnetin-3-O-rutinoside without transformed variables. The last two rows of Table 3 show the sum of $L_{2}$ as a measure for the best overall model and the orders of the models according to $L_{2}$.

**Table 3.** ϵ and $L_{2}$ for all used extracts with Probit, Logit, Log-log, Clog-log, Cauchy and fractional models.

Extract	Model	Probit	Logit	Cauchy	Clog-log	Log-log	Fractional	Optimal
Latex protein	ϵ	0.0440	0.0580	0.1354	0.0967	0.0045	0.0001	-
	$L_{2}$	0.0005	0.0007	0.0053	0.0040	0.0011	0.0101	0.0000
Crude flavonoids	ϵ	-	-	-	-	-	-	-
	$L_{2}$	0.0023	0.0025	0.0106	0.0074	0.0015	0.0006	0.0001
Quercetin with Log transformation	ϵ	-	-	-	-	-	-	-
	$L_{2}$	0.0025	0.0011	0.0571	0.0066	0.0005	0.0014	0.0001
Quercetin without Log transformation	ϵ	-	-	-	-	-	-	-
	$L_{2}$	0.0166	0.0230	0.1328	0.0091	0.0369	0.0364	0.0091
Kaempferol-3-O-rutinoside	ϵ	0.0320	0.0428	0.0995	0.0178	0.0514	0.0001	-
	$L_{2}$	0.0018	0.0003	0.0036	0.0037	0.0009	0.0028	0.0003
Isorhamnetin with Log transformation	ϵ	0.0064	0.0143	0.0656	0.0009	0.0190	0.0001	-
	$L_{2}$	0.0005	0.0001	0.0253	0.0028	0.0003	0.0022	0.0000
Isorhamnetin without Log transformation	ϵ	0.0730	0.0858	0.0545	0.0545	0.0991	0.1011	-
	$L_{2}$	0.0116	0.0170	0.1679	0.0053	0.0241	0.0249	0.0043
The sum of $L_{2}$		0.0358	0.0447	0.4026	0.0389	0.0653	0.0784	0.0139
The order of models		Second	Fourth	Seventh	Third	Fifth	Sixth	First

Table 4 lists values of parameters *a* and *b* for all extracts by all log transformed models. Finally, Table 5 gives concentrations corresponding to 25%, 50% and 75% of FDI for all extracts and all log transformed models.

**Table 4.** The parameters *a, b* for Probit, Logit, Log-log, Clog-log, Cauchy and fractional models.

Extract	Parameters	Probit	Logit	Log-log	Clog-log	Cauchy	Fractional
Latex protein	*a*	0.3061	−7.9182	−4.9567	−6.2157	−9.7138	−0.3984
	*b*	2.5234	4.2561	2.9569	3.0481	5.2076	0.3848
Crude flavonoids	*a*	0.9580	−6.8345	−4.0127	−5.6315	−8.7543	−0.3173
	*b*	1.9423	3.2789	2.1689	2.4608	4.1408	0.2944
Quercetin with Log transformation	*a*	1.3589	−6.4324	−2.9222	−6.0069	−12.274	−0.1789
	*b*	1.4250	2.5346	1.2717	2.2379	5.0502	0.1594
Quercetin without Log transformation	*a*	3.4770	−2.6445	−1.0496	−2.6448	−4.4544	0.0577
	*b*	0.0048	0.0085	0.0044	0.0074	0.0152	0.0005
Kaempferol-3-O-rutinoside	*a*	1.1801	−6.4819	−3.2103	−5.8361	−7.5916	−0.2187
	*b*	1.4737	2.5090	1.3660	2.1272	2.9719	0.1698
Isorhamnetin with Log transformation	*a*	0.0296	−8.7116	−4.0866	−7.9584	−14.941	−0.1978
	*b*	1.8697	3.2945	1.6511	2.8903	5.7822	0.1412
Isorhamnetin without Log transformation	*a*	3.2718	−2.9883	−1.2359	−2.9202	−5.1599	0.0432
	*b*	0.0045	0.0080	0.0041	0.0070	0.0155	0.0004

**Table 5.** Concentrations corresponding to 25%, 50% and 75% FDI using Probit, Logit, Log-log, Clog-log, and Cauchy models.

Extract	Percent	Probit	Logit	Log-log	Clog-log	Cauchy
Latex protein	25%	0.5439	0.5559	0.5111	0.5931	0.6546
	50%	1.0064	1.0072	0.8769	1.1525	1.0185
	75%	1.8624	1.8250	1.7392	1.9456	1.5849
Crude flavonoids	25%	0.7524	0.7798	0.6954	0.8411	1.0359
	50%	1.6739	1.6868	1.4515	1.9151	1.8064
	75%	3.7237	3.6484	3.6920	3.6632	3.1500
Quercetin-3-O-rutinoside	25%	1.6770	1.7663	1.5264	1.8626	2.3720
	50%	4.9872	4.7918	5.3546	4.6034	3.7422
	75%	14.831	12.999	26.317	9.3931	5.9038
Kaempferol-3-O-rutinoside	25%	1.8924	1.9419	1.7938	1.9977	2.2943
	50%	5.4285	5.3222	5.7708	5.1754	4.9789
	75%	15.572	14.586	25.412	10.960	10.805
Isorhamnetin-3-O-rutinoside	25%	2.7565	2.8410	2.6294	2.9183	3.5783
	50%	6.3258	6.1227	6.9128	5.8801	5.3287
	75%	14.517	13.195	23.564	10.214	7.9353

For example, if we want to use Quercetin-3-O-rutinoside to get 75% of FDI we must use the concentration 14.831 mg/mL from the point of view of the Probit model and 12.999 from the point of view of the Logit model.

**Figure 1.**
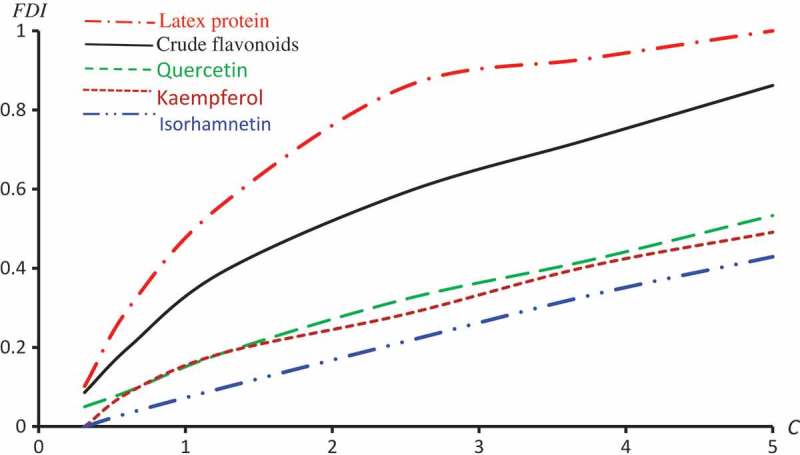
Observed FDI for various concentrations of all extracts.

**Figure 2.**
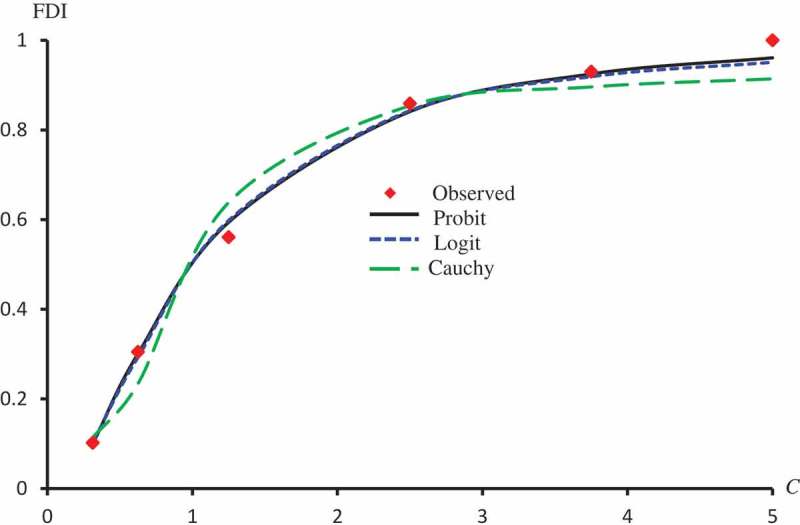
Observed and predicted FDI for various concentrations of Latex protein using Probit, Logit and Cauchy models.

**Figure 3.**
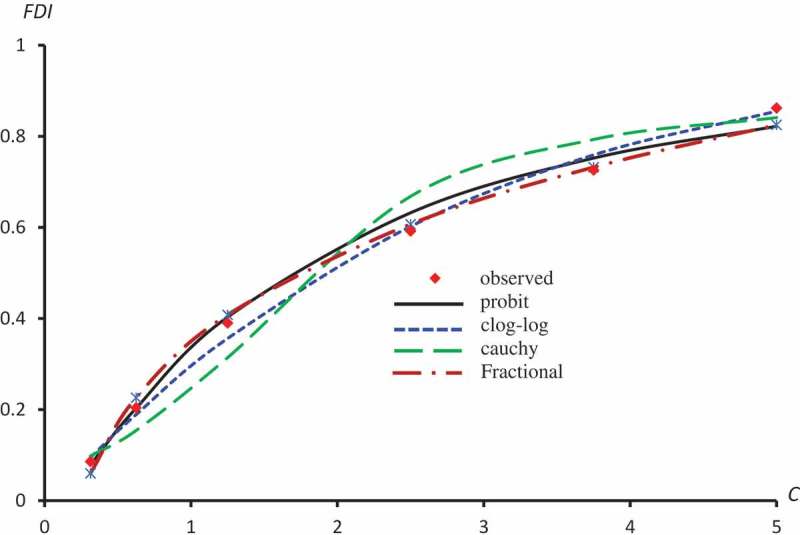
Observed and predicted FDI for various concentrations of Crude flavonoids using Probit, Clog-log Cauchy and Fractional models.

**Figure 4.**
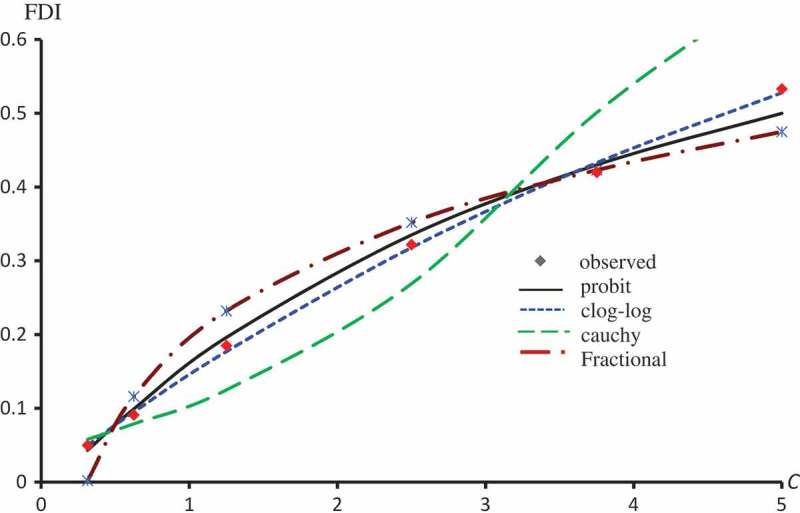
Observed and predicted FDI for various concentrations of Quercetin-3-O-rutinoside using Probit, Clog-log, Cauchy and Fractional models.

**Figure 5.**
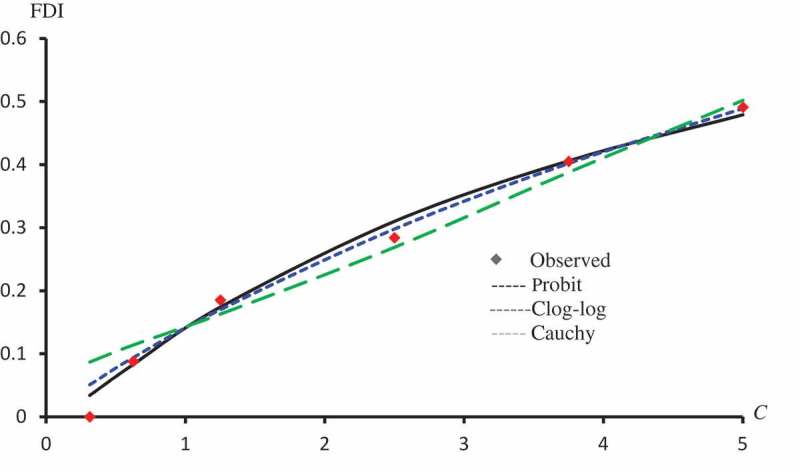
Observed and predicted FDI for various concentrations of Kaempferol-3-O-rutinoside using Probit, Clog-log and Cauchy models.

**Figure 6.**
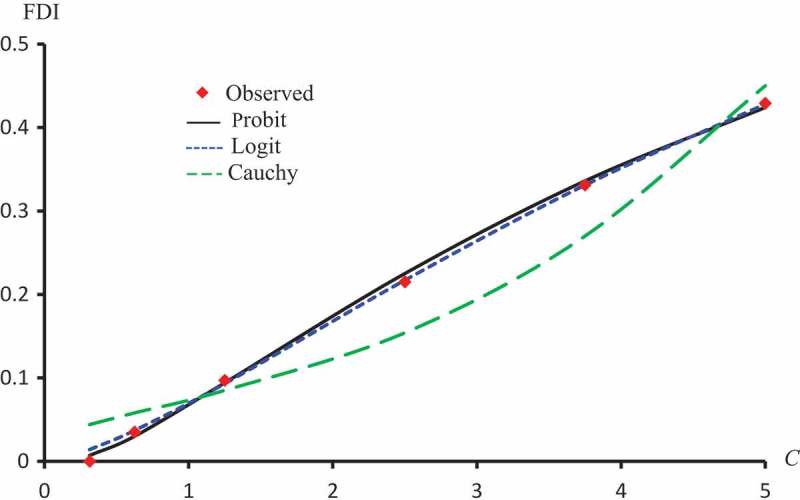
Observed and predicted FDI for various concentrations of Isorhamnetin-3-O-rutinoside using Probit, Logit and Cauchy models.

Figure 1 displays the observed FDI against concentrations for all extracts. Figures (2–6) represent the observed and predicted FDI against concentrations for all extracts and some log transformed models. For these figures, we chose the best log-type model.

**Figure 7.**
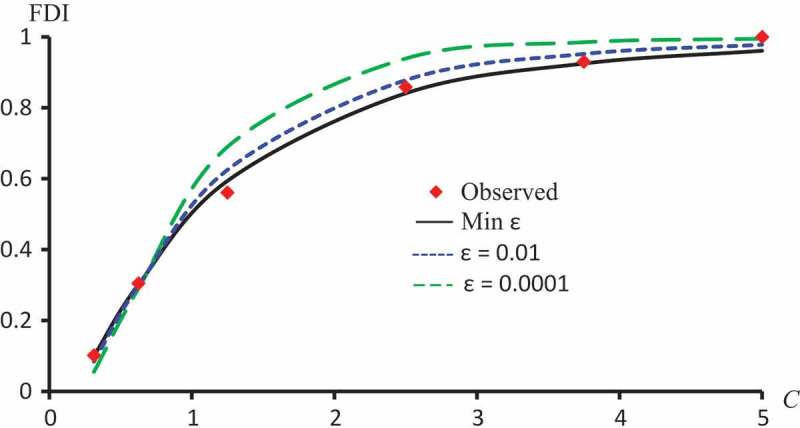
Observed FDI, predicted FDI for minimum $L_{2}$ and predicted FDI with ϵ=0.0001, ϵ=0.01 for various concentrations of Latex protein using Probit models.

**Figure 8.**
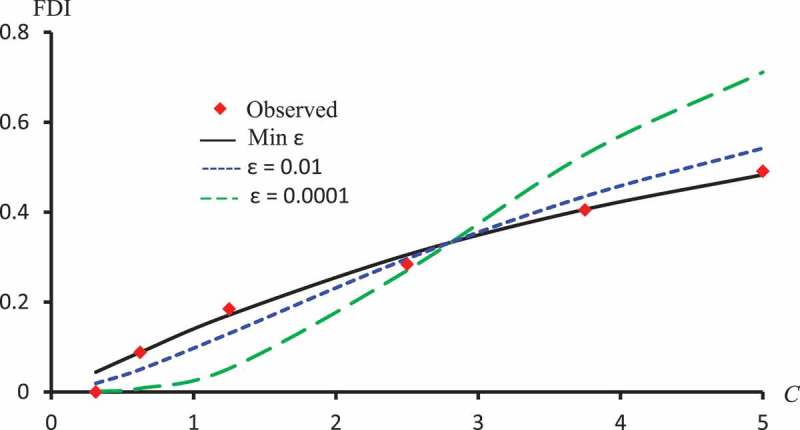
Observed FDI, predicted FDI for minimum $L_{2}$and predicted FDI with ϵ=0.0001 and ϵ=0.01 for various concentrations of Kaempferol-3-O-rutinoside using Logit models.

**Figure 9.**
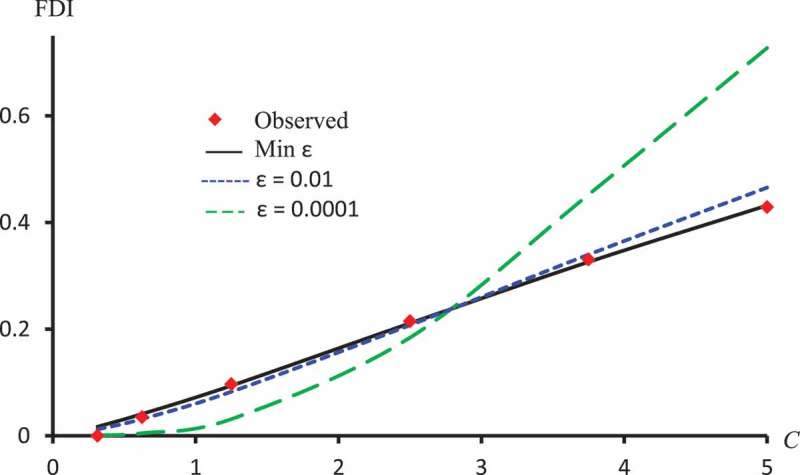
Observed FDI, predicted FDI for minimum $L_{2}$and predicted FDI with ϵ =0.0001 and ϵ=0.01 for various concentrations of Isorhamnetin-3-O-rutinoside using Clog-log models.

Figures (7–9) display observed FDI and predicted FDI curves against concentrations using ϵ=0.01, ϵ=0.0001 and ϵ which minimizes $L_{2}$ for Latex Protein using the Probit model, Kaempferol-3-O-rutinoside using the Logit model and Isorhamnetin-3-O-rutinoside using the Clog-log model respectively.

**Figure 10.**
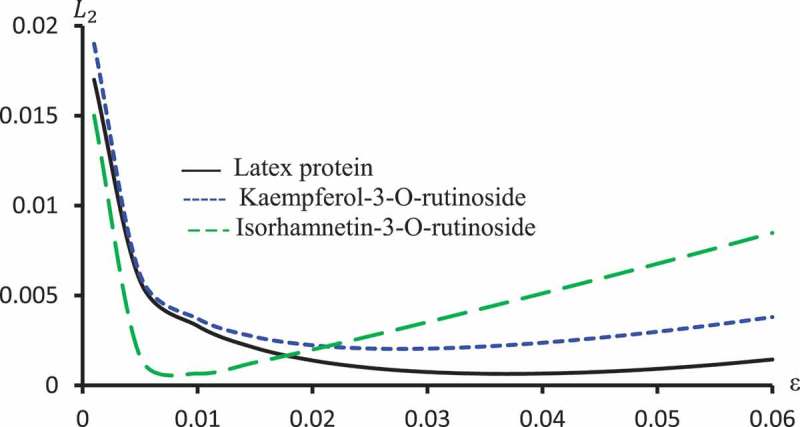
$L_{2}$ against ϵ for Latex protein, Kaempferol-3-O-rutinoside and Isorhamnetin-3-O-rutinoside using Probit models.

**Figure 11.**
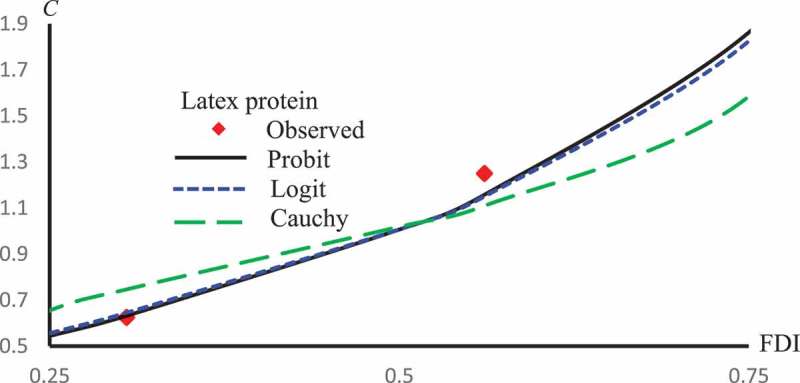
Observed and predicted latex protein concentrations corresponding to FDI using Probit, Logit and Cauchy models.

**Figure 12.**
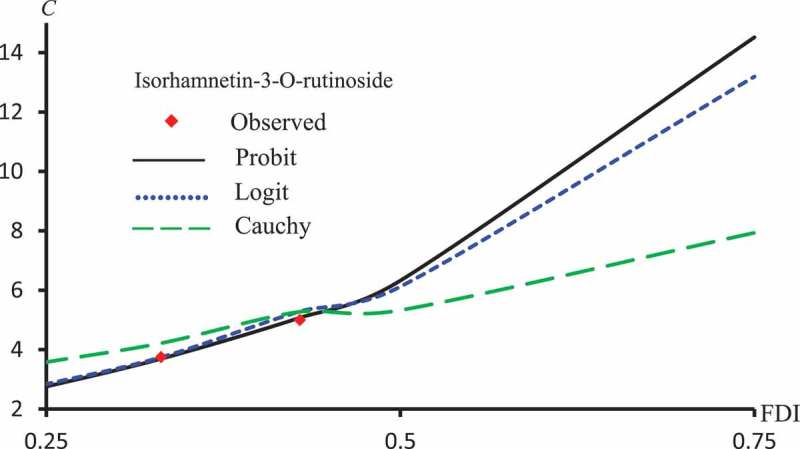
Observed and predicted isorhamnetin-3-O-rutinoside concentrations corresponding to FDI using Probit, Logit and Cauchy models.

**Figure 13.**
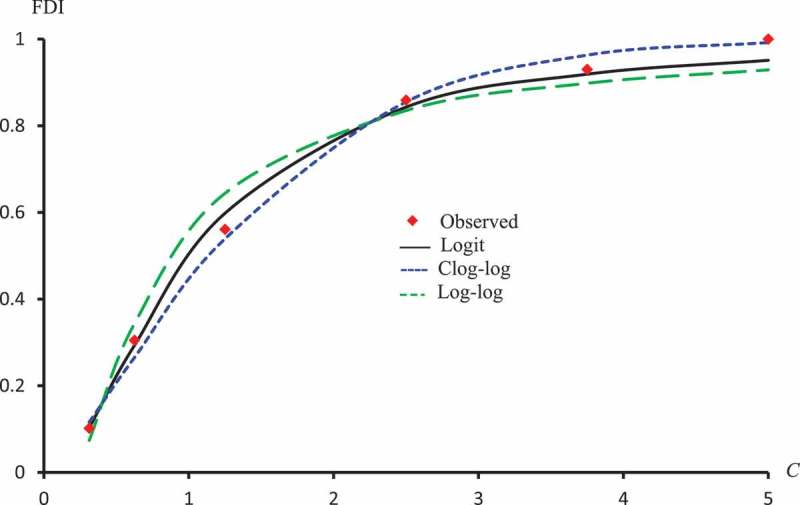
Observed and predicted FDI for various concentrations of latex protein using Logit, Log-log, and Clog-log models.

Figure 10 displays $L_{2}$ against ϵ for Latex Protein, Kaempferol-3-O-rutinoside and Isorhamnetin-3-O-rutinoside using the Probit model. This figure confirms that: it is not the case that the smallest ϵ the smallest $L_{2}$. Figures (11–12) represent concentration-curves against FDI percentages for Latex Protein and Isorhamnetin-3-O-rutinoside using Probit, Logit and Cauchy models. These figures may be considered as a reference for those who want to use concentrations to get a certain percentage of FDI. Figure 13 shows a comparison between the log-type models, i.e., Logit, Log-log, and Clog-log when used to compute the predicted FDI for various concentrations of Latex protein.

## 5 Discussion

Table 3 gives the values of ϵ and $L_{2}$ for all extracts and for all used models with log transformed variables, and also, for Quercetin-3-O-rutinoside and Isorhamnetin-3-O-rutinoside without transformed variables. From this table, it is clear that:

- The models with transformed variables are vastly better than untransformed ones. $L_{2}$ cells of Table 3 confirm this observation. For example, for Quercetin-3-O-rutinoside without log transformation the relative square error, $L_{2}$ with the Probit model is 0.0166 which is approximately 7 times of its correspondence with log transformation (i.e., 0.0025). For the Logit model, the $L_{2}$ value without log transformation is 0.0230 which is approximately 21 times of its correspondence with log-transformation (i.e., 0.0011), for the Cauchy model the $L_{2}$ value without log transformation is approximately twice its correspondence with log transformation, this ratio for Clog-log is approximately 74 times, the same observations noticed for Isorhamnetin-3-O-rutinoside. This conclusion was stated in (Shi and Renton; Alamir et al.,) [11,13], therefore, only results with log transformation are presented and discussed for other models.
- The last column of Table 3 ensures the effectiveness of the optimal procedure explained in 3.7
- The Probit model is the best model for the Latex protein
- The Logit model is the best model for Kaempferol-3-O-rutinoside and Isorhamnetin-3-O-rutinoside.
- The Fractional model is the best model for Crude flavonoids.
- The Clog-log model is the best model for the Quercetin.
- The Log-log model is the best model for Quercetin and Isorhamnetin-3-O-rutinoside without transformed variables.
- The last two rows of Table 3 show the sum of $L_{2}$ for the different models and the orders of the models according to $L_{2}$. As expected, the optimal procedure is the best overall for all extracts. It followed by the Probit model, which followed by the Clog-log model, etc.
- Figures (7–9) clearly demonstrate that the nearest curve to the observed FDI is that obtained with ϵ which minimizes $L_{2}$.

Our results assure that Probit, Clog-log and Logit model FDI with high goodness of fit, one can depend on them to predict the values of *C* and FDI and construct a reliable control system for *R. dominica*.

In a previous study [13], we stated that 2-parameter models are more efficient than 3- and 4-parameter models. Therefore, in this study, we considered the 2-parameter models only.

## Highlights

1. We suggest three models, no study has been used them.
2. Our models develop a precise and long-term strategy to manage the insects.
3. Our strategy leads to fewer hazards to non-target organisms and the ecosystem.
4. To our results, Probit, Clog-log, and Logit model have high goodness of fit.
